# Case of Gastrotomy

**Published:** 1872-12

**Authors:** Francis Troup

**Affiliations:** Auchtermuchty


Case of Gastrotomy (By Francis Troup, M. D., Auchtermuchty: *Edin. Med. Jour.*, July, 1872).—In the year 1867, a man, æt. 50, asked my advice about loss of appetite, and gnawing pain at the epigastrium after taking food. This pain had troubled him more or less for a year, and now vomiting of bloody-looking fluid containing particles of food had set in. No treatment relieved these symptoms; the quantity of food rejected increased; blood was brought up in small nodules, like "boiled liver," as patient described it; and the act of swallowing was slowly performed, and excited a peculiar suffocative cough.

No tumor could be felt, but a bougie passed down the œsophagus encountered a constriction at lower third of sternum; when this was passed, a second, an inch or so lower; and then it found free entrance into the stomach.

During many months, nine at least, attempts were made to keep the passage open by bougies, the patient himself learning to introduce them; then, as increasing difficulty of passing them was experienced, elastic catheters of gradually-diminishing calibre were tried, and a certain amount of food introduced in this way. Nutrient enemata were also freely used. By-and-by the canal became completely closed, and there was constant hawking and coughing up of clear, tenacious, ropy mucus, and for a month before death patient was almost entirely supported by the enemata. During the first part of this month patient complained much of hunger; during the last fortnight, only of a thirst which was something dreadful to bear. He was anxious to live, if possible, or, at all events, to have something done to relieve this tormenting thirst. I carefully explained to him that it was possible to open the stomach and to introduce nourishment; that so doing could not possibly cure him, and might prove fatal; but if he survived, I believed his thirst would cease. He elected to have the operation done; and assisted by the late Dr. David Lyell, of Newburgh, who gave chloroform, and his father, Dr. John Lyell, now of Glasgow, I proceeded to do it in the following manner: A straight incision, three inches long, commencing a little below the extremity of the xiphoid cartilage, was made to the left of the middle line of the abdomen, and midway between it and the costal cartilages. The thin abdominal walls and peritonæum were easily cut through, and, guided by the liver edge, search was made for the stomach,—a somewhat difficult matter, owing to the tenseness of the abdominal parietes, and the shrunken condition of the viscus itself. Pulled down and firmly held by forceps, an opening into it was made, its edges stitched to the parietal incision, and a tracheotomy-tube of moderate size, and with a large shield, secured in the wound. Milk, in gradually increasing doses, and stimulants, were now easily passed into the stomach, and the three remaining days of the man's life were spent in comparative comfort.

The operation scarcely raised the pulse, and the patient repeatedly expressed the opinion that it was worth while having undergone it, were it not for no other thing than the quenching of his thirst. A post-mortem was with difficulty obtained. The cardiac end of the œsophagus was found converted into an epitheliomatous mass, through which, even with the assistance of daylight, no passage could be found. The stomach was very small, and had been struck about its middle. The opposed serous surfaces of stomach and parietal incision were in parts adherent, and the incision itself, in all its length, healing. The peritoneum had not inflamed, — a sufficiently remarkable-fact, considering the free handling to which it had been subjected.—*Philadelphia Med. Times.*

